# How to produce ‘marketable and profitable results for the company’: from viral interference to Roferon A

**DOI:** 10.1007/s40656-019-0268-8

**Published:** 2019-07-30

**Authors:** Carsten Timmermann

**Affiliations:** Centre for the History of Science, Technology and Medicine, The University of Manchester, Simon Building, Room 2.36, Manchester, M13 9PL UK

**Keywords:** Interferon, Cancer therapy, Pharmaceutical industry, Biotechnology, Molecular biology

## Abstract

This paper looks at the commodification of interferon, marketed by Hoffmann La Roche (short: Roche) as Roferon A in 1986, as a case study that helps us understand the role of pharmaceutical industry in cancer research, the impact of molecular biology on cancer therapy, and the relationships between biotech start-ups and established pharmaceutical firms. Drawing extensively on materials from the Roche company archives, the paper traces interferon’s trajectory from observed phenomenon (viral interference) to product (Roferon A). Roche embraced molecular biology in the late 1960s to prepare for the moment when the patents on some of its bestselling drugs were going to expire. The company funded two basic science institutes to gain direct access to talents and scientific leads. These investments, I argue, were crucial for Roche’s success with recombinant interferon, along with more mundane, technical and regulatory know-how held at Roche’s Nutley base. The paper analyses in some detail the development process following the initial success of cloning the interferon gene in collaboration with Genentech. It looks at the factors necessary to scale up the production sufficiently for clinical trials. Using Alfred Chandler’s concept of ‘organizational capabilities’, I argue that the process is better described as ‘mobilisation’ than as ‘translation’.

## Introduction

On 29 March 1960, two young men, Werner Bollag and Jean Lindenmann held a meeting at the Roche factory compound near the river Rhine in Basel, to talk about the potential of a series of laboratory observations to be developed into a marketable drug.^1^ The physician Bollag had been hired by Roche in 1956 to develop and direct the firm’s fledgling cancer research programme. Lindenmann, also in 1956, had started a 1-year visiting fellowship at the National Institute for Medical Research (NIMR) at Mill Hill on the northern edge of London, supported by the Swiss National Academy of Medical Sciences. He studied a phenomenon known as viral interference, collaborating with Alick Isaacs, who ran the World Influenza Centre, a small outfit hosted by the NIMR (On Lindenmann at Mill Hill, see Pieters 2005, pp. 9–32). Lindenmann had come to the NIMR to learn some elementary virus research skills and laboratory techniques, and had not planned to work either with Isaacs or on interference. Lindenmann and Alick are credited with discovering a new substance which in the 1970s was expected to revolutionise the treatment of viral illnesses and cancer: interferon. By 1986, 30 years after Lindenmann’s stay in London, and 26 years after the meeting with Bollag, Roche, as one of a handful of companies world-wide, was able to clinically test, register, mass-produce and finally market a form of interferon developed jointly with the biotech company Genentech under the brand name Roferon-A.

It has become increasingly common over the past two decades to think about the processes that turn scientific observations into medical interventions as translation, and there has been much interest in studying, planning, and streamlining translational research (Austin 2018; Fort et al. 2017). Abandoning the largely discredited linear model of innovation (Godin 2006), but not the desire to develop generalizable statements, recent research on translation has been generating increasingly complex maps of the processes involved (Wagner et al. 2018). Theorists have come to distinguish four phases of translation: T1–T4. T1 encompasses the processes from basic research to initial clinical tests. During T2 effectiveness is established and clinical guidelines are devised. T3 is all about dissemination and implementation. T4, finally, is concerned with outcomes and effectiveness at population level (Fort et al. 2017). In this paper I deal exclusively with T1—a 30-year process in the case of interferon. Moreover, I focus on a specific moment during T1: the transformation of an experimental object into a commodity and the role of industry in this transformation. Many scholars studying innovation rely heavily on sources that allow them insights on basic research and regulatory questions, paying less attention to what happens in industrial contexts. The main reason for this lacuna is not that these aspects are less important, but rather that sources documenting this part of the process are often difficult to access. For this paper I have been relying predominantly on documents in the Roche company archive, complemented by published sources, including the transcripts of oral history interviews with Genentech researchers undertaken by Sally Smith Hughes for her history of Genentech (Hughes 2011). For the early history of interferon I have made heavy use of Toine Pieters’ book (Pieters 2005). The Roferon-A case study, as I will demonstrate, offers new insights on the respective roles of established pharmaceutical companies and new biotech start-ups in what has come to be known as the ‘biotech revolution’.

The story of Roche’s interferon, I suggest, is an excellent case study for understanding the rise of molecular biology and biotechnology in the pharmaceutical industry—a key moment in the recent history of translational medicine. The collaboration between Genentech and Roche illustrates what Martin Kenney has described as the ‘creation of a new economic space’ (Kenney 1998) as outcome of a series of exchanges of personnel, knowledge, practices and funds. The case study demonstrates that new biological medicines such as interferon had their roots not only in collaborations between venture capitalists and university-based molecular biologists around San Francisco and Boston, but also in attempts by traditional pharmaceutical companies to integrate molecular biologists and biotechnological practices with existing research infrastructure and production methods, within existing structures as well as in new institutions, starting in the late 1960s. ‘Disruption’, my case study suggests, may have played a smaller part than is often assumed. Historians of molecular biology and biotechnology may have underestimated the importance of expertise and skills contributed by researchers at industry-funded institutions such as the Roche Institute of Molecular Biology (RIMB) and the Basel Institute for Immunology (BII), founded in the late 1960s and early 1970s. Second, not enough attention has been paid to mundane production technologies and other know-how contributed by traditional pharmaceutical companies.

I will be drawing on a useful framework proposed by the eminent business historian Alfred Chandler in his history of the pharmaceutical industry, focusing on what he terms ‘organizational capabilities’ (Chandler 2005). Chandler distinguishes between ‘technical capabilities’ and ‘functional capabilities’. I will argue that biotech companies such as Genentech had technical capabilities required to successfully establish laboratory processes but lacked the functional capabilities that were needed to commercialise the products of their research in the healthcare market: scaling up production, managing a growing labour force, purchasing raw materials in bulk, dealing with regulatory requirements, and marketing and distribution.

## From interference to interferon

Let’s return to Jean Lindenmann and interferon as an experimental object. In the weeks and months following his arrival at the NIMR in 1956, Lindenmann grew increasingly unhappy with the project he was assigned by his official host and supervisor, Christopher Andrewes, a renowned expert on animal viruses. When he was introduced to Isaacs, who’s Influenza Centre was located in the laboratory next door, Lindenmann and Isaacs discovered that they shared an interest in viral interference, the observation that cells infected by one virus appear to be resistant to infection by a second virus. Neglecting Lindenmann’s official tasks in Andrewes’ lab, they started a series of experiments investigating this phenomenon. They found that interference was a host response to virus infection and not, as most virologists then assumed, a reaction initiated by the first virus to stop the next from taking control of the host cells. Their experiments also demonstrated that interference was mediated by a substance released by the host organism, which they christened ‘interferon’. A series of biochemical tests, undertaken with the help of a biochemist, Derek Burke, suggested that the substance was a protein. Lindenmann presented a paper on his work at the NIMR to the Annual Meeting of the Swiss Society of Microbiology in 1957. This was followed by a piece in the MRC’s Annual Report, a series of two articles in the *Proceedings of the Royal Society of Medicine*, and short announcements in the *Lancet* and the *British Medical Journal*. Over the next few years, Isaacs made contact with a number of other virologists around the globe, who had made similar observations. A small international community of ‘interferonologists’ emerged around these contacts, and was well established by the mid-1960s (Pieters 2005).

The discovery of interferon did not go unnoticed outside the doors of virology laboratories. The British press reported on the ‘viral penicillin’ with some excitement. Drug companies also showed interest. A virologist working for the Basel-based pharmaceutical company Ciba involved Lindenmann in a ‘corridor chat’ following his presentation, but Isaacs did not think it was a good idea to share the manuscript with anyone working for a commercial firm (Pieters 2005, p. 34). He did, however, talk to his bosses at the MRC about the clinical potential of interferon. The MRC filed patent applications in the US, Canada and Germany in 1958, but as more than 6 months had passed since the first publication, they could not obtain a British patent. Following his meeting with Lindenmann at Roche in the spring of 1960, Werner Bollag noted that the lack of specificity in the interferon response was promising and potentially advantageous for developing a chemotherapeutic application against a variety of virus diseases. Currently he felt that basic research was most important. He recommended pursuing the lead with modest means.^2^ However, for the next 10 years, no-one at Roche paid much attention to interferon.

## The old and the new

Roche was a well-established global pharmaceutical company, and its coffers were full in the late 1960s. It had been a trailblazer in the production and sale of synthetic vitamins since the inter-war period, and the tranquilisers Librium and Valium, introduced in 1960 and 1963, were about to top the list of best-selling drugs world-wide for several years (Bächi 2009; Tone 2009). In 1968, the world market in pharmaceuticals—that is sales in pharmacies and hospitals at manufacturer prices—was worth about 10 billion US Dollars. European countries accounted for 40% of this market, and North America for 28%. 26.5% of the revenue was generated in the US alone, where Roche, at its base in Nutley, had a very strong R&D centre. The most important single country for Roche outside the US, generating 16.5% of the income, was Japan. Japan was followed by France with 11%, and Germany and Italy with 8% each. With overall sales of 395 million US Dollars, Roche with its headquarters in Basel and many international bases, was the leading pharmaceutical manufacturer world-wide, followed by the US firms Merck, Sharp & Dohme (MSD) and Lilly. More than half of Roche’s income in the late 1960s came from Librium and Valium.^3^ This was both a blessing and a curse. As an internal memo put it, this uneven distribution of sales presented ‘the well known danger of having too many eggs in one basket and when the basket gets a hole, some or all of the eggs fall out’.^4^

Roche’s management knew that the patents that granted the firm a temporary monopoly on their best-selling drugs were going to expire in the mid-1970s. The unevenly distributed sales profile made the firm vulnerable. Roche had to develop new sources of income. But they also had money to spare to do so. And they were determined to invest some of the money into basic science. As Roche’s then Head of Research, Placidus Plattner stated programmatically in his introductory remarks to a Roche Research Management Group (RRMG) meeting in 1960: ‘Fundamental research is our only source of truly original new products.’^5^ Roche’s investment in a range of basic research initiatives around 1970, including two fully-funded, large institutes, which I will discuss in more detail later, show that this was not just lip service. For now, let’s take a brief excursion back in time, to look at the beginnings of the modern pharmaceutical industry in the second half of the nineteenth century and the place of research and development in its history.

While some companies (like Merck) had roots in older apothecary businesses, more important for the growth of the modern industry and its R&D culture was coal tar chemistry (Aftalion 2001; On the history of the chemical and pharmaceutical industry, see, for example Chandler 2005). Coal-tar was a by-product in the production of coke out of coal and increasingly abundant as steel production took off during the Industrial Revolution. The successful commercialisation of a growing range of coal tar products by chemical factories such as Bayer, Farbwerke Hoechst or BASF started with mass-produced dye stuffs which replaced the traditional, scarce, and hence expensive plant dyes, and found use, above all, in the booming textile industry. The industry employed many of the young chemists trained by pioneers of the new science of chemistry, such as Justus von Liebig. The imagination of synthetic chemists soon extended to medicines, also based on collaborations and joint ventures. Bacteriologists found that they could use such dye stuffs to selectively stain micro-organisms. This made microscopical investigations easier and also led to the idea that such synthetic chemicals could be used to target medicines specifically at these one-celled micro-organisms which had recently been shown to cause many of the most pressing medical problems of the time, such as tuberculosis or syphilis.

The second important pillar supporting the new industry was a patenting system which ensured that companies recovered their investments in research and development. In contrast with traditional patent medicines, the ingredients of the new synthetic medicines were not supposed to be secret, but modern patent laws were developed and implemented to ensure that their production was protected by temporary embargoes, stopping competitors from copying products or processes for a defined period of time (See Dutfield 2003). While the goal was to develop chemotherapies, the first successful products of the collaboration between chemical factories and bacteriologists, however, were not synthetic chemicals but serum products, biologicals such as diphtheria antitoxin, developed and first tested on a human patient by the Robert Koch pupils Emil Behring and Shibasaburo Kitasato in 1891, and commercialised in a joint venture with Farbwerke Hoechst. Companies like Farbwerke Hoechst had capabilities—funds, real estate, technologies and skilled personnel—that could be mobilised in order to turn the observations and ideas of the bacteriologists into practical interventions, standardise them and, importantly, scale up their production.

Many of the new chemical factories were located along the river Rhine in Germany and Switzerland. F. Hoffmann-La Roche & Co. was founded in 1896 in Basel. Unlike its local competitors, Roche immediately focused on the development and production of branded drugs and not dyestuffs or other coal tar products (Peyer 1996). Founded by the son of an old Basel merchant family, Fritz Hoffmann, the company was nevertheless shaped by a chemist, Emil Barell. Initially employed as Hoffmann’s right-hand man, Barell ran Roche from Hoffmann’s death in 1920 onwards until 1952. As was the case in other pharmaceutical companies, research and development at Roche were traditionally dominated by chemists. In 1924, the research department in Basel counted 8 chemists and 1 pharmacologist among its staff, in 1932 it employed 23 chemists and 3 pharmacologists, and after the end of World War II there were 55 chemists and 20 researchers trained as pharmacologists, medical doctors or biologists (Peyer 1996, p. 139). But the dominance of the chemists was about to be challenged in the 1960s, when the traditional approach to pharmaceutical research—screening the effects of a series of substances synthesized by the chemists on laboratory animals and, increasingly, cell culture assays—yielded diminishing returns. A new science, molecular biology, was promising richer pickings and, importantly, the prospect to secure a leading position in the medium to long run.

## Molecular biology

The new science of molecular biology had initially been funded by the Rockefeller Foundation in the interwar period, with the intention to promote a new, reductionist focus in biology on the fundamental particles of life, inspired by the ‘quantum revolution’ in physics (Kay 1993, 2000; Morange 1998). It had attracted non-biologists to biology, including physicists such as Max Perutz, Max Delbrück or Francis Crick. Molecular biologists developed methods that enabled them to study the atomic structures of the macromolecules that made up living organisms: proteins and two types of nucleic acid (best known under the acronyms DNA and RNA). The new science picked up momentum with the discovery of the structure of DNA in 1953, and the deciphering of the genetic code hidden in the sequence of the four organic bases making up DNA, represented by the letters A, C, G and T. It boomed in the 1960s and 1970s, producing increasingly detailed knowledge about the mechanisms of reproduction at the cellular and molecular level: the replication of the strands of DNA in dividing cells and the role of RNA, the other nucleic acid, in translating genetic information into proteins and transporting it to the places in the cell where these proteins were produced. Molecular biologists developed elaborate toolkits for manipulating DNA and RNA, and by the early 1970s it had become possible to conceive of a world where human genes and bacterial DNA could be ‘recombined’, and human proteins produced by bacteria in fermenters. These recombinant DNA techniques were a source of excitement as well as concern, leading to a voluntary moratorium in 1975, following a conference in Asilomar, California, which involved many of the leading pioneers of the new science (Krimsky 1982).

Molecular biology carried great promises for medicine. The new science promised not only a key to a molecular understanding of illness and, thus, drugs that targeted disease-causing malfunctions in the body—such as cancer—at their molecular roots. Recombinant DNA technology also created the prospect of new production methods for biological molecules with therapeutic potential. While pharmaceutical companies in their R&D departments adopted the analytical tools created by molecular biology relatively quickly, as they hoped that this would help them to develop a more targeted approach to drug development, it is often assumed that they were reluctant to embrace recombinant DNA technology, leaving this field to small start-up companies financed by venture capital, springing up around universities, especially in California and around Boston (Bud 1994; Hughes 2011; Rasmussen 2014; Teitelman 1989; Wright 1994). Partly this was due to the continuing dominance of chemists, it has been argued, and partly simply to conservatism (Rajan 2006). Robert Teitelman has characterised the US drug companies as ‘the prototypical Republicans of corporate America: large, rich, and very conservative’ (Teitelman 1989, p. 141). The Roche archives tell a slightly different story, of more sustained investment in molecular genetics research and its applications, and interferon was at the centre of this story.

## RIMB and BII

Roche’s research managers were aware of the new science, and they wanted in. They expected that a fertile exchange between ‘traditionally capable chemists’ and ‘a group of exceptional molecular biologists’ would provide the company with opportunities to establish ‘a unique “Long Range Approach” in the development of new drugs’.^6^ But how would they find these molecular biologists? And more importantly: how would they persuade them to work for a pharmaceutical company? An often-held assumption was that they didn’t, and that this partly explained the success of the biotech start-ups. One big advantage of these start-ups was their closeness to universities, both geographically and in terms of work culture. For young, talented post-docs who liked the work culture they had grown accustomed to during their PhDs, and who were keen on a career in research, the move from a university lab to a biotech start-up was far easier than that to the R&D department of one of the established pharmaceutical companies. In fact, in the early days this may not even have involved a physical move, as spin-off companies operated from inside university labs. However, there were indeed ways by which molecular biologists could be persuaded to work for old pharma.

Roche’s response to the challenge of persuading molecular biologists to work for the company was to fund research institutes at or near its headquarters, the Roche Institute of Molecular Biology (RIMB) in Nutley and the Basel Institute for Immunology (BII). Both emulated the work culture and, importantly, the freedom of university labs and government-funded research institutes such as the US National Institutes of Health, to pursue projects because of their intellectual rather than commercial interest, and to publish the results.^7^ Scientists did not, in the first instance, work to improve the balance sheet of the company; they worked on promoting their careers and reputations as scientists. The benefits for the company were expected to be long-term and indirect. The reputational gains would reflect positively onto the company sponsoring the institutes, and promising results could be shared with the R&D department, developed, and commercialised. Post-doctoral training programmes would provide the company with access to highly trained but modestly paid, motivated workers, who did not expect permanent positions but could be recruited for jobs in R&D if they had the right skills (Bürgi 2011).

The Nutley plans took shape very quickly, helped by a series of contingencies. Some accounts suggest that the very idea of launching the institute was the result of a conversation between friends and former laboratory colleagues at a cocktail party in Bethesda in 1967: Sidney Udenfriend, then head of a laboratory at the US National Institutes of Health (NIH) and John Burns, the new Vice President for Research at Roche Nutley. Udenfriend, a protein biochemist had joined the laboratory of the future NIH Director, James Shannon in the 1950s, during a time of rapid growth, which saw the Bethesda institutes transform into a major centre of biomedical science (Weissbach and Witkop 2003). Burns had been a colleague of Udenfriend’s during their time as postgraduate research students at New York University in the 1940s. Their idea of launching a Roche-funded basic science institute was enthusiastically supported by Roche Nutley’s President, Virginius D. Mattia, a medical doctor. Udenfriend also knew Alfred Pletscher, Roche’s Director of Research based in Basel, trained both as a chemist and medical doctor, who had spent a year at the NIH as a research scientist in the mid-1950s to get up to speed with the latest methods in biochemistry (Weissbach 1989, p. 233). Roche managers reached agreement with a group of NIH researchers who were willing to leave Bethesda for the planned Roche Institute, many working with Udenfriend, who was going to be the RIMB’s Director.^8^

The RIMB started operating in the same year from an office in Bethesda. Mattia signed a Charter, confirming that the institute ‘will be wholly devoted to long-range basic research designed to shed light on fundamental life processes’ (Weissbach 1989, p. 238). The Charter granted the scientists at the Institute ‘independence in their choice and pursuit of research problems, guided by the scientific importance of a project.’ The institute was going to have a Board of Advisors. Researchers were going to be ‘encouraged to accept appointments at local universities and to participate in university teaching’. There was also going to be a postdoctoral training programme and a programme for visiting scientists (Weissbach 1989). Until laboratory space became available in Nutley, the researchers who had committed to joining the RIMB and signed a contract with Roche, were encouraged to remain at their present locations or spend time as visiting scientists in other laboratories. Building work for the RIMB started on the edge of the Nutley campus in September 1968. Before they moved into the new building in May 1971, RIMB labs were temporarily accommodated in existing buildings. This prepared the ground for productive interactions between RIMB researchers and Roche research scientists which, according to Weissbach’s history of the RIMB, ‘provided an important basis for future communication’ (Weissbach 1989, p. 245).

One of the researchers making the move from Bethesda to Nutley was a former member of Marshall Nirenberg’s research staff, Sidney Pestka. Nirenberg, an expert on protein synthesis completed ground breaking work on deciphering the genetic code with Heinrich Matthäi in the 1960s, for which he was awarded a Nobel Prize in 1968. He had been Udenfriend’s lab neighbour at the NIH and was a member of the RIMB Advisory Board. Pestka was interested in the mechanisms by which different antibiotics inhibited protein synthesis. At the National Cancer Institute, the Special Cancer Virus Program was gaining momentum (Scheffler 2014, 2019; Zoon 2017). Pestka had developed an interest in the molecule that a decade earlier had been hailed as viral penicillin: interferon. He was going to play a central role in the molecular characterisation of interferon and in its cloning a few years later.^9^

Developing the Basel-based institute was slightly more challenging. Pletscher felt that state-funded and university-based research in relevant areas was not very well developed in Switzerland, and that it was difficult to identify institutions and researchers to work with. The choice of a suitable focus for the new institute was going to be informed by a range of issues: first, the research area had to open up new possibilities to tackle medical and therapeutic problems which had not been accessible by conventional means. Second, the area needed to have achieved a certain degree of maturity, so that results could be expected. Third, qualified researchers and technical staff needed to be available, and fourth, there had to be potential for collaborations with existing institutions. An area that fulfilled these criteria was immunology. Immunological processes played a role in conditions such as cancer, virus diseases, arthritis, and control of the immune system was important for organ transplantations. Immunology was also attractive because of its close links with cell and molecular biology.^10^

By 1968, the plans for the BII were well advanced (Lefkovits 2017). A site had been chosen and prepared not far from the main Roche campus in Basel, the architect’s drawings were done and, most importantly, a director had been appointed.^11^ The chosen candidate, was Professor of Immunology at Frankfurt and Director at the Paul Ehrlich Institute (Söderqvist 2002, 2003). By 1970, the first part of the new building was completed and in operation, and by 1971 the whole institute.^12^ The intention was to run the institute according to the same principles as the RIMB, fully-funded by Roche but on a very long leash, and overseen by an eminent and international Advisory Board. Like the RIMB, the BII was home to research that turned out to be useful for the commodification of interferon. As I will discuss in more detail later, monoclonal antibodies developed at the Institute were employed in the purification procedure, and in biological assays, used to monitor production and in clinical trials. Two BII scientists, Georges Köhler and Niels Jerne were awarded a Nobel Prize in 1984, jointly with César Milstein at the MRC Laboratory for Molecular Biology at Cambridge, for the development of the monoclonal antibody technique (Cambrosio and Keating 1995; Marks 2015).

## Interferon and the war on cancer

While there was much support among Roche’s senior management for funding basic science, it was clear that ultimately this had to feed into new products. ‘It is all very well, even refreshing and entertaining to a certain degree, that we are meeting twice a year discussing research matters, “chewing the fat” on a scientific level’, the chemist Dr Max Furter, a member of Roche’s *Generaldirektion* stated at the Roche Research Management Group meeting in 1957. ‘However, the most important part of our duty and the main task on hand never to be forgotten for a second is to produce results, and I mean marketable and profitable results for the Company.’^13^ A decade later, there was also some scepticism about the promises of the new molecular biology for a company where chemists had traditionally produced reliable results. As one of Roche’s research managers, Otto Isler put it in 1969 (employing contemporary gender stereotypes): ‘chemistry is at Roche’s disposal just like a good wife: reliable, cooperative, maybe sometimes smiling with the knowledge that everything which had been realized came to a very great extent from her.’ Isler warned fellow research managers: ‘As you all know, Roche-earnings are due to sales of substances in the form of specialties or bulk and seldom or never in the form of theories or speculations.’^14^ So did interferon look like a substance likely to generate revenues?

The initial excitement over the prospect of finding a viral penicillin, in fact, had calmed down fairly quickly. Pieters speaks of a ‘vote of no confidence in the clinical potential of interferon as a therapeutic drug’ (Pieters 2005, p. 89). Nevertheless, a small community of dedicated ‘interferonologists’ continued research on viral interference throughout the 1960s, exchanging samples of interferon extracts generated in different experimental systems and working towards common standards and a shared nomenclature. They also explored interferon’s effects on tumours in experimental animals, finding that interferon inhibited the growth of tumours in mice and thus suggesting that it may have potential as a new medicine against human cancers (Pieters 2005, pp. 111–116). The interferon community included the Finnish virologist Kari Cantell, who struck a deal with the Red Cross Blood Transfusion Service in Helsinki, allowing him and his colleagues to extract human interferon from leukocytes fractionated off as a by-product from donor blood. The amounts produced by this method were sufficient for small-scale clinical trials. Initial tests of the clinical efficacy of this interferon were under way by the early 1970s in Sweden, conducted by an associate of Cantell’s, Hans Strander, at the Karolinska Institute, on a small number patients suffering from advanced osteogenic sarcoma, whose disease had failed to respond to other treatments (Panem 1984, p. 14). However, Sandra Panem in her book on *The Interferon Crusade* argues that the most important boost for interferon’s transformation from a potential viral penicillin into a possible cancer drug was a campaign spearheaded by Mathilde Krim, a Swiss-born physician working at the Memorial Sloan-Kettering Cancer Center (Panem 1984). Krim was an associate of the influential anti-cancer lobbyist and philanthropist Mary Lasker. An alliance led by Lasker was also the main force behind US President Richard Nixon’s declaration of War on Cancer in 1971 (Patterson 1987, pp. 248–251).

Documents in the Roche archives confirm that the transformation of interferon into a saleable commodity was getting under way around the same time. Roche research managers revisited the therapeutic potential of the elusive substance in 1971. John Burns at Roche Nutley introduced an Interdisciplinary Research Meeting on antiviral and anticancer research on 21 May 1971 as follows:

In light of the Government’s major commitment to anticancer research, Roche Research – internationally based and uniquely equipped in facilities and expert manpower – could contribute to this endeavour by expanding our experience in this field into a broad research programme.’^15^

Roche, it seems, was mobilising for Nixon’s War on Cancer, and work on interferon was thought to be integral to the planned programme. Roche Nutley was starting work on a coordinated cancer programme, which was expected to overlap with its antiviral programme and coordinated with projects at the RIMB. In addition to classical screening, this would include, according to Burns, ‘new approaches to anti-tumor testing and studies on the isolation, structure and mode of action of interferon’.^16^ Interferon was to be a vehicle for introducing a new direction in the rational development of anti-cancer drugs. And indeed, by the early 1980s, interferon was the spearhead of a whole research programme on immunomodulators.^17^ Immunolators created some excitement among medical researchers: these substances mediated the immune system and thus seemed to promise a path to natural cures for a whole range of diseases, including cancer (Löwy 1996).

Anti-cancer drugs, up to this point, had not made anyone much money, so this was not an obvious move for a large pharmaceutical firm used to blockbuster drugs, although the declaration of war on cancer made it more likely. Sales of anti-cancer drugs accounted for only 0.1% of the total world market in pharmaceuticals in 1966 (11.21 million US Dollars in absolute terms), projected to increase to 0.3% by 1971 (37.12 million Dollars).^18^ By the 1980s, Bristol-Myers was the US market leader in anticancer chemotherapy, selling five out of the top ten anticancer drugs. This generated 150 million Dollars in sales and gave Bristol a 40% share of the market. This was still small fish, however, compared to blockbuster drugs which were approaching a billion Dollars in sales. However, the market for anti-cancer drugs was growing by about 25% per year from the early 1970s (Teitelman 1989). One reason for this steady growth, as a Roche Oncology Task Force Report stipulated in 1986, was ‘the market’s relative lack of sensitivity to high cancer therapy costs and drug price increases’.^19^ In 1976, in fact, at 58% plus, cytostatics (drugs that stop cells from dividing) registered the largest growth of all therapeutic groups.^20^ Roche had two cytostatics in its programme, both developed as part the broader chemotherapy research programme (including antibacterial chemotherapies) at Nutley: 5-Fluorouracil (or 5-FU), synthesised in 1956, and Natulan (procarbazine), first marketed in 1964.^21^ Neither contributed much to Roche’s overall sales income.

Around 1970, the established therapies for most tumours involved combinations of surgery and radiation, while cancer chemotherapy was still relatively new and experimental (Löwy 1996; Pickstone 2007). Tumours tended to develop resistances against drugs fairly quickly, and many doubted whether the few months survival time gained were worth the often drastically unpleasant side effects. The gruesome experimental combination chemotherapies, which by the mid-1970s stopped cancers in children for long enough to be considered cures, were controversial and viewed as unethical by many in the US, and even more so in Europe (Barnes 2007; Keating and Cambrosio 2012; Krueger 2008). Interestingly, however, ambiguity over the value of chemotherapy in the treatment of cancer turned interferon into an even more attractive proposition.

Most early anti-cancer drugs developed from the 1940s to the 1970s were designed to tackle cancer by interfering with the metabolism of cells that divided rapidly. In this way they killed cancer cells and made tumours shrink, but the chemotherapy also affected all other tissues in the body where growth and cell division happened: the follicle cells at the roots of hairs, for example, or the cells in the lining of the mouth which produced the lubricants for saliva. The new combination chemotherapies delivered several salvoes of toxicity, one after the other, meant to kill the dwindling numbers of cancer cells that survived the previous drug, and in this process often nearly killed the patient. Interferon was different: assumed to be non-toxic (wrongly, as it turns out), it was part of the natural immune response against viruses and expected to activate the body’s natural defences against cancer cells. This was an attractive proposition at a time of growing environmental sensitivities, which saw people become increasingly sceptical about the physiological effects of chemicals (Löwy 1996, p. 125). But before interferon could be promoted as a real alternative, it had to be properly purified and characterised, and a way of producing amounts sufficient for full-scale clinical trials had to be developed—a process that needed resources.

## Mobilising organizational capabilities

Panem suggests that a conference that Mathilde Krim organised in the spring of 1975 to raise interest in interferon especially at the National Cancer Institute (NCI) was a watershed moment in her crusade. However, this notion has been disputed by Pieters, who characterises Krim’s plans as a ‘house of cards based almost entirely on Strander’s 14 cancer patients’ (Panem 1984, pp. 16–20; Pieters 2005, p. 128). He finds that representatives of government agencies and companies who attended the event were not particularly impressed by the evidence presented and that the press almost ignored the event (Pieters 2005). Arguably, the observation of viral interference had been ‘translated’ successfully into an understanding that interference was mediated by a substance, interferon, with the potential to control tumour growth by enhancing the response of the immune system to cancerous cells. Interferon at this point was not conceptualised as ‘immunotherapy’—this only happened in the mid-1980s, when recombinant interferon was about to be marketed. Crucially, interferon as an actual, pure substance still did not exist. Cantell’s human interferon was a relatively crude extract, and the characterisation of its effects was work in progress.

In order to turn interferon into a clinically useful, sufficiently pure substance for routine use, resources and capabilities were needed—financial, technical, and human: people with the necessary expertise and contacts. Purification methods had to be developed and refined. Production needed to be scaled up in stages, first to provide enough interferon for clinical trials, and subsequently further in order to bring the substance to market. A New Drug Application (NDA) needed to be prepared and submitted. Regulatory approval had to be sought and communication channels maintained with the FDA in the United States and regulatory bodies in other countries. I argue that the resources that could be mobilised by companies such as Roche, Schering-Plough and Wellcome, who all marketed different interferons in the mid-1980s, were absolutely crucial in this story. And importantly, these companies were not new biotech start-ups, but established pharmaceutical firms who had, over decades, acquired what the business historian Alfred Chandler calls ‘organizational capabilities’, both technical and functional (Chandler 2005, pp. 6–9). Genentech and other biotech start-ups had no established organisational culture in the 1970s. While they had what Chandler terms ‘technical capabilities’, relating to the R in R&D, they almost completely lacked the ‘functional capabilities’ they needed to commercialise the products of their research in the healthcare market. The start-ups lacked know-how, for example on scaling up, the management of a growing labour force, the purchasing of raw materials, marketing and distribution, customers and markets and, importantly for medicines and diagnostic kits: regulatory frameworks. Without the initial investments made by established pharmaceutical companies, there were not even going to be large-scale clinical trials. I will now take a closer look at how Roche’s and Genentech’s organizational capabilities were employed to bring a type of interferon to market.

## Cloning interferon

In 1978, Sidney Pestka and his colleagues at the RIMB succeeded in their attempts to purify human interferon. Once purified, the next step was to determine its amino acid sequence. Once this had been achieved, too, fragments of the peptide were to be synthesized and tested for biological activity. Finally, as the minutes of a Research Management Group meeting in 1978 state: ‘The eventual production of human interferon through recombinant DNA technology [was] considered a real possibility.’^22^

Nicolas Rasmussen characterises recombinant human interferon as ‘the genetic engineer’s top prize: exceedingly rare, exceedingly valuable, sure to win fame, and sure to attract the toughest competition’ (Rasmussen 2014, p. 53). Recombinant DNA technology was still new in 1978, and the biotech boom that was unfolding around the promise of producing unlimited amounts of human proteins and other therapeutically active biological substances in bacteria was in its early stages. On 6 September 1978, the Chief Executive of Genentech, the most successful of the new biotech start-up companies, announced that scientists at the firm had successfully cloned the gene encoding human insulin in Escherichia coli bacteria. Insulin was the first success in the race for recombinant versions of therapeutically useful macromolecules, a peptide that had started its career as therapeutic substance more than five decades earlier in form of a brown extract prepared by Frederick Banting and his student Charles Best in a laboratory at the University of Toronto, similarly impure as the interferon preparations from Finland.

However, in contrast with interferon, there was no shortage of good quality insulin produced by other means, as Rasmussen argues: ‘Rather, the scientists picked insulin. For the multiple entrepreneurial biologists who simultaneously decided to clone this protein hormone, it represented the project best suited for exploiting a newly emerging congruence of business and scientific interest’ (Rasmussen 2014, p. 40). Rasmussen also points out that, once it was successfully cloned, Genentech needed a partner to turn their insulin into a pharmaceutical. Lilly, one of the big American pharmaceutical firms developed the production techniques and took care of the regulatory procedures required to bring recombinant insulin to market (Rasmussen 2014, pp. 66–67).

Following the young company’s insulin success, by 1979 Roche was negotiating with Genentech about collaborating on the cloning of interferon. Working with Genentech, the Roche negotiators conceded in a memo dated 28 May 1979, was:

the only efficient and realistic solution. After the initial (outrageous) demands and conditions put forth by Genentech, an agreement has now been reached which seems to serve the interests of both parties in a balanced and satisfactory way.^23^

However, there was no intention to rely on Genentech forever:

we see the collaboration with Genentech only as the unavoidable but most effective crutch for the interferon project and not as a permanent dependence of Roche for future developments involving recombinant DNA technology.^24^

In May 1980, a Laboratory for Recombinant DNA Research (LRDR) was created at Nutley, and the Roche research managers agreed that all of the company’s research centres should acquire some expertise in these new technologies.^25^

In effect, Roche commissioned Genentech with the cloning of interferon and agreed to pay the biotech firm for using the interferon-producing bacteria so generated. The contract between the two companies states:

GENENTECH will grant certain rights to ROCHE and will, with ROCHE’s financial and other support, conduct certain research and development aimed at the creation and delivery of said organism, and that ROCHE’s payment therefor will be determined as a royalty for use over a set term.^26^

Roche’s ‘financial and other support’ was substantial, despite an obvious reluctance on Pestka’s part to help the Genentech team around David Goeddel too proactively (Goeddel 2003; Rasmussen 2014, pp. 103–106). Pestka’s team in Nutley tried to clone interferon independently, supplying Goeddel with the same mRNA they were using, which was derived from a cell line Pestka had been given by a former NIH colleague, Robert Gallo, who in turn had received it from David Golde, a haematologist and oncologist working at the University of California, Los Angeles (UCLA). Samples were traded freely, then, between laboratories, in an informal gift exchange economy without much thought about commercial exploration. It helped that Pestka did not work directly for Roche but was based at the RIMB, which by then had acquired an excellent reputation for its research.^27^ Using a biological assay he had developed, Pestka managed to clone and isolate a cDNA fragment encoding part of the interferon gene. Using this fragment as a probe, Goeddel isolated a cDNA sequence long enough to carry the whole interferon gene. Roche and Genentech had their interferon clone. They were not alone: Biogen, a Boston-based start-up headed by a Swiss professor, Charles Weissmann, and collaborating with Schering-Plough, had also successfully cloned an interferon gene.

The race was over for the molecular biologists when the ‘gene jockeys’ (Rasmussen’s term) had succeeded in producing their clones, but the real work for the Roche researchers at Nutley was only starting. More resources were needed to carry the project forward. The infrastructure had to be created for scaling up fermentation and purification processes. By September 1980, research managers at Roche believed that they had a ‘lead over competitors’.^28^ They expected that by December of the same year, sufficient material was going to be available to start preclinical development. Clinical trials were scheduled to start in spring 1981. Plans for phase I and II trials were put in place for studies in viral diseases and cancer. Production was initially slow due to the limited size of the fermenters approved for use in the project. Genentech obtained permission to use larger fermenters, and Roche Nutley applied for permission in January 1981. Roche was aiming to maintain independence from Genentech by expanding its expertise in production and purification. When updated about the details, Roche’s Chief Executive, Fritz Gerber ‘agreed that the interferon project should be assigned top priority for development.’^29^

## Developing interferon

I discuss the development process here in considerable detail as this illustrates the importance of existing organizational capabilities at Nutley and Basle. Cloning a human interferon gene into bacteria was only the first step towards producing recombinant interferon. The bacteria had to be grown, and the interferon had to be extracted and purified. The use of recombinant DNA techniques had to conform with strict NIH rules, following the end, in June 1976, of the moratorium which molecular biologists led by Paul Berg had imposed on themselves at Asilomar in February 1975 (Krimsky 1982, 2005). When interferon production was scaled up for clinical trials in 1981, changes to processes and equipment, including the use of larger fermenters still had to be reviewed by a dedicated biosafety committee and approved by the NIH (Wright 1994).

The purification process developed at Nutley was another example of Roche benefiting directly from earlier investment in basic research, in this case both at the RIMB and the BII: researchers at Nutley developed a process involving monoclonal antibodies developed in Basel by colleagues who had been transferred from the BII to Roche. These antibodies, attached to the matrix of a High Pressure Liquid Chromatography (HPLC) column performed the crucial step in the isolation of interferon from the other constituents of the bacterial cell extract (Pestka 2007). The antibodies specifically bound interferon, while everything else simply ran through the chromatography column. A change of solvent subsequently released the interferon. Besides the purification columns, antibodies developed in Basel for the different types of interferon were also used in radio immunoassays (RIA) and enzyme immunoassays (EIA). These assays helped to characterise the interferons (it turned out that there were several ‘species’ of interferon) and monitor interferon levels during production and in pharmacokinetic studies.^30^ The antibodies had been developed by the Applied Immunology Project group headed by Theophil Staehelin at Roche Basel. Staehelin had been one of the first researchers recruited for the new BII in 1970, where he provided important expertise in molecular biology. Alfred Pletscher, Roche’s Head of Research had convinced the Roche management to launch the Applied Immunology Project in 1977 with 22 dedicated positions. This followed a meeting with Niels Jerne, Staehelin and others, convened to discuss the article manuscript—still unpublished—in which Georges Köhler and César Milstein discussed their new hybridoma method of producing monoclonal antibodies (Lefkovits 2017, pp. 128–129). Pletscher persuaded Staehelin to move down the road from the BII to Roche, where the latter assumed the formal position of a Vice-Director (Lefkovits 2017, p. 129).

The Interferon project group at Nutley worked hard to prepare the submission of an Investigative New Drug (IND) application for Recombinant Leukocyte Interferon alpha (IFL-rA) to the FDA before the Christmas holidays, on 23 December 1980. The IND application had to be approved before any investigational drug could be administered to humans—the precondition for clinical trials to start. A toxicology study was carried out over the holidays in squirrel monkeys. The researchers noted no significant adverse responses; they did not observe the depression of white blood cells noted in earlier studies with impure recombinant leukocyte A interferon obtained from Helsinki. Through informal contacts the Roche researchers knew by 7 January that a letter authorizing the initiation of clinical studies on 15 January 1981 had been typed and was about to be mailed.^31^

## Scaling up: ‘these matters are treated as crash programs’^32^

The first of two clinical studies to evaluate tolerance and pharmacokinetics was undertaken under the auspices of Jordan Gutterman at the MD Anderson Cancer Center in Houston, Texas. A member of the Nutley team, Dr Fein personally carried an interferon sample to Houston and stayed there to witness the initiation of the studies. A second study was carried out at Stanford University under the auspices of the virologist Thomas Merrigan. These Phase I drug escalation studies were expected to take about 10 weeks and finish towards the end of March. Plans were being prepared for the multi-dose studies, most likely multi-center, to be initiated following the completion of the Phase I studies.^33^

Still in January, a member of the Nutley team, Dr Makover visited Staehelin’s group in Basle to learn how to produce monoclonal antibodies.^34^ The so-called hybridoma method involves fusing antibody-producing B cells with immortal myeloma (tumour) cells in what is known as a hybrid cell line (Marks 2015). The resulting hybridoma cell culture has the potential to produce unlimited amounts of the desired antibody. The quickest method to produce these antibodies was the so-called ascites method: the hybridoma cells were injected into the abdominal cavity of a mouse, where fluid accumulated over a week or two, which contained the antibody. This fluid was then ‘harvested’ and the antibody isolated. Each mouse produced about 30 mg of purified antibody. To meet the requirements for purifying interferon for the initial, small-scale clinical trials, at least 300 mice were needed. For each gram of purified interferon, the researchers calculated that they required approximately three grams of antibody. To produce 1 kg of purified IFL-rA per year, thus, they would need 65,000–85,000 mice—a considerable logistical challenge, which may have also raised ethical questions.^35^ Different procedures for the production of antibodies in cell culture, without the need for mice, were also explored.^36^

The project group estimated that they needed approximately 10 g of IFL-rA for the Phase II studies, and 100 g for Phase III. By 1985, they projected that approximately 6 kg of IFL-rA were required per year, based on the assumption that three clinical indications were going to be approved. This meant that either additional fermentation capacity at Roche would have to be made available, or bacteria had to be engineered that produced larger amounts of interferon, or both.^37^ By March 1981, planning had started for a move to bigger fermenters—from 10 L to 100 gallons. But this move had implications, both administrative and practical. In order to use the bigger fermenters, suitable methods had to be found and developed for killing the bacterial cells. Several different approaches were being explored (one of them recommended by Genentech). Importantly, if plants and practices were modified, there was no guarantee that the final product was identical with the one submitted for the IND application. The Roche researchers, thus, had to make sure that the end product still met IND specifications.^38^

Meanwhile the clinical trials were delivering the first results. 14 cancer patients had entered the escalating single dose studies designed to determine tolerance and pharmacokinetic parameters. Three patients had to discontinue as they appeared to reach the maximum tolerated dose, experiencing substantial fatigue and flu-like symptoms. These symptoms appeared to be dose related, and were known from the trials with Cantell’s human interferon from Helsinki. This was a bit disappointing, as researchers had initially hoped that recombinant interferon would not cause these side effects. A meeting was scheduled with FDA officers for 30 March 1981, to give them a status report. Plans were also made to start clinical studies in Japan and Europe, using the IND approval granted to Roche Nutley Roche’s International Research Coordination and Advisory Group decided at its March 1981 meeting that ‘Roche as a whole should participate in this new development’.^39^ Capacities for the production of monoclonal antibodies were to be established also in Japan, and a workshop on recombinant DNA techniques was going to be held in Nutley, involving representatives from all Roche centres. As recombinant interferon generated more and more publicity, a policy was needed for the distribution of samples to researchers. Genentech and Roche agreed that they would inform each other prior to the distribution of material for pre-clinical research. Genentech appears to have dealt with such requests more restrictively, and investigators who had been denied material from Genentech, in several cases then contacted Roche and received it. The Interferon Research Steering Committee decided to provide limited amounts of material to investigators ‘who we feel are competent and whose results would compliment [sic] our overall research interest in this field’.

There were also some technical problems not related to fermentation and purification that had to be taken care of: in March 1981, Dr Goldberg of Nutley’s Pharmacy R&D department alerted the members of the Interferon Steering Committee of the need for additional capacity to handle the vialing of IFL-rA in preparation for the Phase II and III trials. Moreover, freeze drying capacity was limited, and there were potential staff shortages as other development programmes competed with interferon, despite the high priority that was given to this project.^40^ These technical challenges were also met, drawing on what Chandler characterises as ‘technical capabilities’. These were not related to research but to production and the rather mundane handling and packaging of finished products, an area where Roche’s experience far outweighed Genentech’s. By June 1981, sufficient amounts of interferon were available to take the clinical testing of recombinant interferon to the next stage.^41^

## Trialling interferon

The beginning of the clinical trials in 1981 meant that Roche were the first company undertaking such trials with a biotechnologically produced cancer drug. Plans were also under way to start production and prepare clinical trials in Europe and Japan. Interferon provided Roche research managers with a focus for discussing and planning improvements to the international coordination of such trials, with a view to streamlining drug applications in different countries. ‘Effective and efficient utilization of our worldwide clinical facilities is an important aspect of the drug development process’, the minutes of a meeting in March 1981 note, ‘and was the subject of a lengthy discussion.’^42^

Roche’s main competitors in the US were Schering in collaboration with Biogen for IFL-rA, Cetus and Shell for fibroblast interferon and Genentech for immune interferon. Schering and Biogen were producing in very large fermenters, but were experiencing problems with gene expression and purification. They they did have, however, a very intensive programme for clinical trials, with 5 full-time physicians in the US assigned exclusively to the oncology clinical programme. They also appeared to pay clinical centres two to three times as much per patient as Roche did. In the UK, Wellcome was very active with their leukocyte interferon, subsidised in part by the government.^43^

By March 1982, several trials in oncology and virology were under way, pursued with high priority. In the US, phase II studies in breast cancer and lymphoproliferative diseases were being initiated. Protocols for phase II breast and lung cancer studies had been approved by the Medical Protocol Review Committee. Research managers also believed that it was necessary to start thinking about using interferon in combination with chemotherapy and radiation.^44^ The collaboration with Genentech was scheduled to end. Roche managers felt that at the RIMB and the new Molecular Genetics Department at Nutley they had enough practical expertise to go it alone. At its meeting in September, the Pharma Research Committee recommended that the firm concentrated on a small number of clinical indications: lymphoma, myeloma and Kaposi sarcoma.^45^ Ironically, however, with the finishing line in sight, the project ran into trouble.

IFL-rA was not the only immunomodulator developed by Roche in the early 1980s. Parallel programmes were running for a whole family of interferons, including recombinant interferons alpha D, beta and gamma, and fibroblast interferon, along with a small zoo of other immunomodulators, including interleukin-1 and -2 and several thymosins. The declared goal was to develop a whole range of these products, but coordinating this complex programme was increasingly challenging. Meanwhile, not even the compound most advanced in its development was ready to go on sale: IFL-rA, which Roche were planning to market as Roferon-A. In fact, not even the Product Licence Application had been submitted.

A Pharma Research Committee meeting held in April 1984 at the Berkshire Place Hotel in Midtown Manhattan focused on strategic questions rather than reviewing individual projects. The minutes state the problem very clearly. It was no longer that there were too few eggs in the basket:

Currently the most critical factor with a negative impact on our Pharma business and morale is the slow pace of development of identified product candidates. The problem is not confined to the question of whether we can cope with such a large number of development products in clinical trial but extends into basic issues whether Roche as an organization can afford to be present in such a large number of therapeutic areas.^46^

Following extensive discussions over when a project could be considered ‘ripe’ for development, the group decided to come up with a plan for a more focused Roche research and development programme (the minutes use the term ‘concentration’). Albert Hürlimann, Roche’s overall Head of Research and Cedric Hassall, Head of Research at the company’s British HQ in Welwyn, expressed concern that ‘in certain areas Roche moved too far into non-targeted basic research’, and that these areas were not ‘ripe’.^47^ How ripe were the many immunomodulators that Roche researchers were working on? A Product License Application (the equivalent of a New Drug Application, NDA, for biologicals) would soon be filed in the US for Roferon-A, for Kaposi’s sarcoma and malignant melanoma. ‘The NDA is weak,’ the committee conceded, ‘and, therefore, Basle has agreed to use these data for filing an NDA in only 5 countries.’^48^ Preclinical research on other interferons was terminated, and only the preclinical research in support of Roche’s and Genentech’s clinical trials of the combination of Roferon-A with recombinant interferon gamma was allowed to continue.^49^

Roferon A was finally approved by the FDA in June 1986, initially for hairy cell leukaemia, and later for chronic myelogenous leukemia, non-Hodgkin’s lymphoma, malignant melanoma, and Kaposi’s sarcoma. ‘Although only approved for a small number of cancer types,’ comments the report of an Oncology Task Force appointed by the Roche management:

it is anticipated that alfa interferon will be used widely, in both clinical treatments and clinical trials, by itself and in combination with other types of interferon and cytotoxic drugs, outside this range of approved tumors.^50^

## Conclusion

Finally available as a marketed medicine, 30 years after Lindenmann’s stay at the NIMR, and 26 years after his meeting with Werner Bollag, interferon did not turn out to be the miracle drug that many had hoped for. It was no blockbuster. The trial results for the handful indications for which Roferon A was approved in 1986 were good, but for common ‘solid’ cancers such as breast, colon or lung cancer—the big killers—results were disappointing.^51^ Toine Pieters argues that the companies marketing interferon successfully positioned the substance as what he calls ‘a “helpful neighbour” compatible with and supportive of existing treatment practices’ (Pieters 1998). Rather than being administered as a single agent, interferon was included in a growing number of combination treatment regimes, both experimental and routine, for a variety of conditions. However, rather than one of many immunomodulators as initially envisaged, Roferon A remained the only compound of this type in Roche’s product portfolio. While it did not bring the expected immunomodulator revolution in cancer therapy and the treatment of viral disease, though, it marked the beginning of a different kind of transformation, much slower but no less significant for the company. By the early twenty-first century, Roche was the leading producer world-wide of cancer medicines, with a portfolio of targeted cancer drugs and major investments in diagnostic technologies, wholeheartedly embracing the ideas associated with the label ‘personalised medicine’ (Hedgecoe 2004; Tutton 2014). Many of these new products were monoclonal antibodies developed for therapeutic purposes, an idea that Roche research managers had been considering in the early 1980s, such as rituximab (MabThera), trastuzumab (Herceptin) or bevacizumab (Avastin).

While developing recombinant human interferon alpha into Roferon A, a declared goal of Roche’s research managers was to not depend on Genentech in the future, but acquire the expertise needed to produce protein compounds with recombinant DNA technology in-house. They were also considering the use of recombinant proteins as starting points for a new approach in chemical synthesis, with the aim of simulating the actions of these proteins. The reality looked very different. The collaboration with Genentech continued. Moreover, by 1990 a majority of Genentech shares were owned by Roche, and in 2008 Roche took over the San Francisco-based biotech company completely. Genentech’s San Francisco campus became Roche’s US HQ, and Nutley was closed in 2012. In 1997 Genentech launched a ‘BioOncology Initiative’, as part of the preparation for the market introduction of rituximab and trastuzumab—both were marketed by Genentech in the US and by Roche in the rest of the world. The first product marketed under the ‘BioOncology initiative’, however, was Roferon-A (‘Genentech Focuses On Cancer as it Launches BioOncology Initiative’ 1997).

How do we best characterise the relationship between Roche and Genentech in the 1970s and early 1980s? While Alfred Chandler in his broad-brush history of the pharmaceutical industry gets some of the details wrong, his conceptual framework of what he calls ‘organizational capabilities’ is useful when it comes to thinking about this relationship. As I have argued in this paper, Genentech and other biotech start-ups had no established organisational culture in the 1970s. While they had what Chandler labels ‘technical capabilities’, relating to the R in R&D, they almost completely lacked the ‘functional capabilities’ required to commercialise the products of their research in the healthcare market: know-how on scaling up production, managing the labour force, purchasing raw materials, marketing and distribution, customers and markets and, importantly for medicines and diagnostic kits: regulatory frameworks. This led to some spectacular failures in the biotech boom of the 1970s and 1980s (Teitelman 1989), and continues to cause problems as the recent case of Theranos shows, a start-up that aimed to disrupt the lucrative market for diagnostic tests, whose founders not only ignored regulatory requirements but actively sought to subvert them (Carreyrou 2018). The biotech start-ups imported aspects of their management culture from venture capital companies, but more important was the influence of the work culture in universities and research institutes, especially in the United States. This had advantages and disadvantages. One big advantage was that the closeness of the start-ups to universities, both geographically and in terms of work culture, made it much easier to recruit young talent willing to self-exploit. However, when they wanted to bring their products to market, biotech start-ups had to collaborate with pharmaceutical firms. In effect, the biotech companies acted as extended laboratory benches for the pharmaceutical houses, which also benefited from the fact that the risks of failure attached to the early stages of the R&D process were covered by investors who held stock in the start-ups. Mirowski and Horn in their study of contract research organisations focus on companies running clinical studies (Mirowski and Horn 2005). My Roferon-A case study suggests that the early relationship between Roche and Genentech may present another example of contract research.

Roche had embraced molecular biology in the late 1960s to prepare for the moment when the patents on its bestselling drugs were going to expire. The company funded two basic science institutes to gain direct access to talents and scientific leads. These investments, I have argued, were crucial for Roche’s success with recombinant interferon, along with more mundane, technical and regulatory know-how held at Roche’s Nutley base. As I have shown, however, the company overstretched in the early 1980s when attempting to develop a whole family of immunomodulators for market introduction. As a consequence, research managers felt they had to concentrate efforts, but they were unsure about how to identify a project that was ripe for development. Faced with the realisation that fundamental research conducted in-house did not necessarily deliver the desired leads, Roche management decided to close down both the RIMB (in 1997) and the BII (in 2001), to the regret of the scientific community. The departure from basic research was part of a broader change of direction in research management in the pharmaceutical industry, described, for example, in a book by Jürgen Drews, who joined Roche as Head of Pharma Research in 1985 and retired from the company as global Head of Research in 1998 (Drews 2003). Writing in 1998, Drews outlines a future in research and development where pharmaceutical companies focus exclusively on development and marketing, leaving all research to biotech companies and, increasingly, universities. The takeover of Genentech, appears to contradict this strategy, as Roche merged with Genentech in the US and Genentech managers joined the Roche Board in Basel. Clearly, Roche dows not fit the image presented by Kaushik Sunder Rajan in his much-cited book, *Biocapital*, where he assumes that ‘big pharmaceutical companies still tend to rely for the most part on the development of small therapeutic molecules through organic chemical synthesis’ (Rajan 2006, p. 23). Roche, in effect, has turned into what may be best described as a ‘biotech giant’, and interferon was the catalyst.

## Footnotes

1 Memo, 29 March 1960, Roche Historical Archives (hereafter RHA), PD.3.1.RFA 103270i. Memos and minutes in the Roche Archives are either in English or German. Translations of literal quotes from German are mine.

2 Bollag Memo, 29 March 1960, RHA, PD.3.1.RFA 103270i.

3 Memo, Dr K. Feinstein, Betrifft: Pharmazeutischer Weltmarkt 1968, RHA, FE.0.4 102852.

4 Ibid. Spelling and punctuation as original.

5 Minutes of the RRMG meeting in Gottlieben, Switzerland, 25–30 April 1960, RHA, FE.0.4 101129a.

6 Minutes of the RRMG meeting in Princeton, New Jersey, 11–16 June 1970, RHA, FE.0.4 101129c.

7 One of the explicit goals of the new institutes was ‘to enhance Roche’s reputation in the scientific world’. Minutes of the RRMG meeting, 5–8 June 1968, RHA FE.0.4 103593d.

8 A. Pletscher, Notes for RRMG Meeting, 30 May 1967, RHA, FE.0.4 102852.

9 Pestka himself tells the story in a film produced to promote Roche’s research, ‘The Quest’ (1979). RHA BT.1.1.9 – 400190. Given the promotional nature of the film, this source has to be handled with care. However, Pestka’s central role in the cloning of interferon is corroborated by Goeddel (2003).

10 A. Pletscher, Notes for RRMG Meeting, 30 May 1967, RHA, FE.0.4 102852.

11 Minutes of the RRMG meeting, 5–8 June 1968, RHA FE.0.4 103593d.

12 Minutes of RRMG meetings, 11–16 June 1970 and 10–15 June 1971, RHA FE.0.4 101129c.

13 Minutes of the RRMG meeting, 24–30 April 1957 in Brestenberg, Switzerland. RHA FE.0.4 101129a.

14 RHA, FE.0.4 103593h.

15 Minutes of the Antiviral Research/Anticancer Research meeting on 21 May, 1971, included with papers for 1971 RRMG meeting. RHA, FE.0.4. 103593i.

16 Minutes of the RRMG meeting, 10–15 June 1971. Hotel Lygon Arms, Broadway. RHA, FE.0.4 101129c.

17 Minutes of the Pharma RCM, 11–12 September 1980. RHA, FE.0.4 107293b. A workshop in 1981, designed to ‘bring the knowledge and expertise at the Basle Insitute of Immunology to the closer attention of Roche scientists’, focused on immunomodulators. IRCAG Meeting, 23–24 March 1981. RHA, FE.0.4 107293c.

18 Papers for the RRMG meeting, 11–16 June 1970. RHA FE.0.4 103593f.

19 Oncology Task Force Report, p. 8. RHA, FE.0.4 105780b.

20 RRMG meeting, Rigi, Switzerland, 16–18 June 1977. RHA, FE.0.4 101129d.

21 5-FU formed the basis for a number of successful topical treatments for skin cancer and other skin diseases, including psoriasis. Roche has continued to invest in the 5-FU: Xeloda (capecitabine), one of Roche’s more recent cancer drugs is converted into 5-FU in the body. Procarbazine was one of the two ‘P’s in MOPP, a combination chemotherapy regimen developed at the NCI by a team around Vincent de Vita. Roche stopped producing and marketing Natulan in 1997, selling the rights to the drug to a former Roche manager who launched his own company.

22 Minutes of the RRMG meeting, 27–30 June 1978, London. RHA, FE.0.4 107293b.

23 Memo, 28 May 1979. RHA, PD.3.1.RFA 107156.

24 Ibid.

25 Minutes of a meeting of the International Research Coordination and Advisory Group (hereafter IRCAG), 13–14 September 1980, and of discussions with Mr Gerber, Mr Lerner and some members of General Management on 15 September. RHA, FE.0.4 107293b.

26 Draft contracts in RHA, PD.3.1.RFA 107156.

27 UCLA sued Roche in 1980 for unauthorised use of the cell line, and they settled in 1982 to avoid bad publicity. Golde himself was subsequently sued by the patient whose cells were used (Rasmussen 2014, p. 117).

28 Minutes of a meeting of the IRCAG, 13–14 September 1980, and of discussions with Mr Gerber, Mr Lerner and some members of General Management on 15 September. RHA, FE.0.4 107293b.

29 Minutes of a meeting of the IRCAG, 13–14 September 1980, and of discussions with Mr Gerber, Mr Lerner and some members of General Management on 15 September. RHA, FE.0.4 107293b.

30 Interferon Research Steering Committee and Interferon Clinical Development Committee, Minutes of Meeting on 7 Jan 1981. RHA, FE.9.3 103555a.

31 Interferon Research Steering Committee, Meeting on 7 Jan 1981. RHA, FE.9.3 103555a.

32 Minutes, IRCAG Meeting, Nutley, 23–24 March 1981, p. 7. RHA, FE.0.4 107293.

33 Interferon Research Steering Committee, Meeting on 7 Jan 1981. RHA, FE.9.3 103555a.

34 Interferon Research Steering Committee, Meeting on 19 Jan 1981. RHA, FE.9.3 103555a.

35 Interferon Research Steering Committee, Meeting on 19 Jan 1981. RHA, FE.9.3 103555a.

36 Interferon Research Steering Committee, Meeting on 11 March 1981. RHA, FE.9.3 103555a.

37 Interferon Research Steering Committee, Meeting on 19 Jan 1981. RHA, FE.9.3 103555a.

38 Interferon Research Steering Committee, Meeting on 11 March 1981. RHA, FE.9.3 103555a.

39 Minutes, IRCAG Meeting, Nutley, 23–24 March 1981. RHA, FE.0.4 107293c.

40 Interferon Research Steering Committee, Meeting on 19 Jan 1981. RHA, FE.9.3 103555a.

41 Interferon Research Steering Committee, Meeting on 5 June 1981. RHA, FE.9.3 103555a.

42 Minutes, IRCAG Meeting, Nutley, 23–24 March 1981. FE.0.4 107293c; Minutes, Pharma-RCM 1981, Basle, 10–12 September 1981, p. 180. RHA, FE.0.4 107293c.

43 Minutes, IRCAG Meeting, Nutley, 22–23 March 1982. RHA, FE.0.4 107293c.

44 Minutes, IRCAG Meeting, Nutley, 22–23 March 1982. RHA, FE.0.4 107293c.

45 Minutes, Pharma RCM, Nutley, 20–21 September 1982. RHA, FE.0.4 107293c.

46 Minutes, Pharma RCM, New York City, 12–13 April 1984. RHA, FE.0.4 107293d.

47 Ibid.

48 Ibid.

49 Minutes, IRCAG Meeting, Basle, 28–29 October 1985. RHA, FE.0.4 107293d.

50 Oncology Task Force Report, p. 53. RHA, FE.0.4 105780b.

51 Clinical Research Status Report, Ro 22-8181, Roferon R-A, Interferon Alfa-2A, January 1985. RHA, PD.3.1.RFA.

## References

[CR1] Aftalion F (2001). A history of the international chemical industry.

[CR2] Austin CP (2018). Translating translation. Nature Reviews Drug Discovery.

[CR3] Bächi B (2009). Vitamin C für alle! Pharmazeutische Produktion, Vermarktung und Gesundheitspolitik (1933-1953).

[CR4] Barnes E (2007). Between remission and cure: Patients, practitioners and the transformation of leukaemia in the late twentieth century. Chronic Illness.

[CR5] Bud R (1994). The uses of life: A history of biotechnology.

[CR100] Bürgi M (2011). harmaforschung im 20. Jahrhundert: Arbeit an der Grenze zwischen Hochschule und Industrie.

[CR6] Cambrosio A, Keating P (1995). Exquisite specificity: The monoclonal antibody revolution.

[CR7] Carreyrou J (2018). Bad blood: Secrets and lies in a silicon valley startup.

[CR8] Chandler AD (2005). Shaping the industrial century: The remarkable story of the evolution of the modern chemical and pharmaceutical industries.

[CR9] Drews J (2003). In quest of tomorrow’s medicines: An eminent scientist talks about the pharmaceutical industry, biotechnology, and the future of drug research.

[CR10] Dutfield G (2003). Intellectual property rights and the life science industries: A twentieth century history.

[CR11] Fort DG, Herr TM, Shaw PL, Gutzman KE, Starren JB (2017). Mapping the evolving definitions of translational research. Journal of Clinical and Translational Science.

[CR12] Godin B (2006). The linear model of innovation: The historical construction of an analytical framework. Science, Technology and Human Values.

[CR13] Goeddel, D. V. (2003). *Scientist at Genentech, CEO at Tularik* (S. S. Hughes, Ed.). https://oac.cdlib.org/view?docId=kt467nb7wh&brand=oac4&doc.view=entire_text. Accessed May 3, 2018.

[CR14] Hedgecoe A (2004). The politics of personalised medicine: Pharmacogenetics in the clinic.

[CR15] Hughes SS (2011). Genentech: The beginnings of biotech.

[CR16] Kay LE (1993). The molecular vision of life: Caltech, the Rockefeller Foundation, and the rise of the new biology.

[CR17] Kay LE (2000). Who wrote the book of life? A history of the genetic code.

[CR18] Keating P, Cambrosio A (2012). Cancer on trial: Oncology as a new style of practice.

[CR19] Kenney M, Thackray A (1998). Biotechnology and the creation of a new economic space. Private science: Biotechnology and the rise of the molecular sciences.

[CR20] Krimsky S (1982). Genetic alchemy: The social history of the recombinant DNA controversy.

[CR21] Krimsky S (2005). From Asilomar to industrial biotechnology: Risks, reductionism and regulation. Science as Culture.

[CR22] Krueger GM (2008). Hope and suffering: Children, cancer, and the paradox of experimental medicine.

[CR23] Lefkovits I (2017). History of the Basel Institute for Immunology.

[CR24] Löwy I (1996). Between bench and bedside: Science, healing, and interleukin-2 in a cancer ward.

[CR25] Marks L (2015). The lock and key of medicine: Monoclonal antibodies and the transformation of healthcare.

[CR26] Mirowski P, Horn RV (2005). The contract research organization and the commercialization of scientific research. Social Studies of Science.

[CR27] Morange M (1998). A history of molecular biology.

[CR28] Panem S (1984). The interferon crusade.

[CR29] Patterson JT (1987). The dread disease: Cancer and modern american culture.

[CR30] Pestka S (2007). The interferons: 50 years after their discovery, there is much more to learn. The Journal of biological chemistry.

[CR31] Peyer HC (1996). Roche: Geschichte eines Unternehmens, 1896-1996.

[CR32] Pickstone JV (2007). Contested cumulations: Configurations of cancer treatments through the twentieth century. Bulletin of the History of Medicine.

[CR33] Pieters T (1998). Marketing medicines through randomised controlled trials: The case of interferon. BMJ: British Medical Journal.

[CR34] Pieters T (2005). Interferon: The science and selling of a miracle drug.

[CR35] Press Release. (1997). Genentech Focuses On Cancer as it Launches BioOncology Initiative. New BioOncology initiative works to commercialize pipeline of biotherapeutic anti-cancer products. https://www.gene.com/media/press-releases/4851/1997-05-15/genentech-focuses-on-cancer-as-it-launch. Accessed December 20, 2018.

[CR36] Rajan KS (2006). Biocapital: The constitution of postgenomic life.

[CR37] Rasmussen N (2014). Gene jockeys: Life science and the rise of biotech enterprise.

[CR38] Scheffler RW (2014). Managing the future: The Special Virus Leukemia Program and the acceleration of biomedical research. Studies in History and Philosophy of Science Part C: Studies in History and Philosophy of Biological and Biomedical Sciences.

[CR39] Scheffler RW (2019). A contagious cause: The American hunt for cancer viruses and the rise of molecular medicine.

[CR40] Söderqvist T (2002). The life and work of Niels Kaj Jerne as a source of ethical reflection. Scandinavian Journal of Immunology.

[CR41] Söderqvist, T. (2003). *Science as autobiography: The troubled life of Niels Jerne* (D. M. Paul, Trans.). New Haven: Yale University Press.

[CR42] Teitelman R (1989). Gene dreams: Wall Street, academia, and the rise of biotechnology.

[CR43] Tone A (2009). The age of anxiety: A history of America’s turbulent affair with tranquilizers.

[CR44] Tutton R (2014). Genomics and the reimagining of personalized medicine.

[CR45] Wagner J, Dahlem AM, Hudson LD, Terry SF, Altman RB, Gilliland CT (2018). A dynamic map for learning, communicating, navigating and improving therapeutic development. Nature Reviews Drug Discovery.

[CR46] Weissbach H, Drews J, Melchers F (1989). Reflections on the Roche Institute of Molecular Biology after 20 years. Forschung bei Roche/Research at Roche: Rückblick und Ausblick/Reminiscences and Reflections.

[CR47] Weissbach, H., & Witkop, B. (2003). Sidney Udenfriend. April 5, 1918–December 29, 1999. In *National Academy of Sciences, Biographical Memoirs* (Vol. 83, pp. 270–299). Washington, DC: The National Academies Press. 14983827

[CR48] Wright S (1994). Molecular politics: Developing American and British regulatory policy for genetic engineering, 1972-1982.

[CR49] Zoon KC (2017). Sidney Pestka (1936–2016). Journal of Interferon and Cytokine Research.

